# Natural Killer Cells and Neuroblastoma: Tumor Recognition, Escape Mechanisms, and Possible Novel Immunotherapeutic Approaches

**DOI:** 10.3389/fimmu.2014.00056

**Published:** 2014-02-12

**Authors:** Cristina Bottino, Alessandra Dondero, Francesca Bellora, Lorenzo Moretta, Franco Locatelli, Vito Pistoia, Alessandro Moretta, Roberta Castriconi

**Affiliations:** ^1^Dipartimento di Medicina Sperimentale, Università degli Studi di Genova, Genova, Italy; ^2^Istituto Giannina Gaslini, Genova, Italy; ^3^Dipartimento di Onco-Ematologia Pediatrica, Ospedale Bambino Gesù, Roma, Italy; ^4^Università di Pavia, Pavia, Italy; ^5^Centro di Eccellenza per le Ricerche Biomediche, Università degli Studi di Genova, Genova, Italy

**Keywords:** neuroblastoma, natural killer cells, PVR, B7-H3, TGF-beta, tumor escape mechanisms, immunotherapeutic approaches, chemokine receptors

## Abstract

Neuroblastoma (NB) is the most common extra-cranial solid tumor of childhood and arises from developing sympathetic nervous system. Most primary tumors localize in the abdomen, the adrenal gland, or lumbar sympathetic ganglia. Amplification in tumor cells of MYCN, the major oncogenic driver, patients’ age over 18 months, and the presence at diagnosis of a metastatic disease (stage IV, M) identify NB at high risk of treatment failure. Conventional therapies did not significantly improve the overall survival of these patients. Moreover, the limited landscape of somatic mutations detected in NB is hampering the development of novel pharmacological approaches. Major efforts aim to identify novel NB-associated surface molecules that activate immune responses and/or direct drugs to tumor cells and tumor-associated vessels. PVR (Poliovirus Receptor) and B7-H3 are promising targets, since they are expressed by most high-risk NB, are upregulated in tumor vasculature and are essential for tumor survival/invasiveness. PVR is a ligand of DNAM-1 activating receptor that triggers the cytolytic activity of natural killer (NK) cells against NB. In animal models, targeting of PVR with an attenuated oncolytic poliovirus induced tumor regression and elimination. Also B7-H3 was successfully targeted in preclinical studies and is now being tested in phase I/II clinical trials. B7-H3 down-regulates NK cytotoxicity, providing NB with a mechanism of escape from immune response. The immunosuppressive potential of NB can be enhanced by the release of soluble factors that impair NK cell function and/or recruitment. Among these, TGF-β1 modulates the cytotoxicity receptors and the chemokine receptor repertoire of NK cells. Here, we summarize the current knowledge on the main cell surface molecules and soluble mediators that modulate the function of NK cells in NB, considering the pros and cons that must be taken into account in the design of novel NK cell-based immunotherapeutic approaches.

## Neuroblastoma, Where are We?

Neuroblastoma is a very heterogeneous disease (1) that includes rare familial (<2%) and sporadic forms. It presents as a locoregional (Stage 1, 2, and 3 or L1, L1/L2, and L2) or progressing metastatic disease involving bone, skin, liver, brain, and bone marrow (BM), the latter being frequently refractory to standard therapy (Stage 4 or M). Moreover, a rare metastatic form of spontaneously regressing/maturating disease (Stage 4S or MS) may occur in children below the age of 18 months (2–5). Over the years, major efforts have been focused to unveil the genetic and biological features of the different forms of NB in order to identify novel prognostic factors and druggable targets. To date, stage, patient’s age, and presence or absence of the amplification of MYCN, a transcription factor crucial for central nervous system (CNS) development (6), are considered major predictors of patients’ clinical outcome. Moreover, risk-group stratification is based upon additional parameters such as loss of chromosome 11q, tumor histology, and ploidy (3, 5). MYCN amplification strongly associates with loss of heterozygosity at chromosome 1p and occurs in approximately 15% of children affected by NB, with increased frequency in stage 4 patients (approximately 50%). A more accurate evaluation of the risk of treatment failure according to these prognostic factors allowed overtime a significant reduction of mortality in patients that at diagnosis presented with localized non-MYCN-amplified tumors (low-risk) and non-MYCN-amplified stage 4 under 18 months of age (intermediated-risk), who received less aggressive surgical and chemotherapeutic treatments. In particular, Rubie and coworkers have recently demonstrated that low-dose chemotherapy improved 5 years survival of 9% in infants with low-risk NB without threatening symptoms compared to cases treated with high dose chemotherapy due to resistance to the low-dose regimen or presence of one threatening symptom (7). A more dramatic scenario exists in children with high-risk NB at diagnosis, which includes stage 4 patients <18 months with MYCN amplification or >18 months with or without MYCN amplification and rare patients with MYCN-amplified localized tumors (3, 5). These patients have a 5-year survival rate <50% although receiving aggressive combination therapies that include intensive chemotherapy, surgery, radiotherapy, autologous stem cell transplantation, and the administration of Retinoids (13-*cis*-retinoic acid) (3, 5), which represent adjuvants for high-risk NB therapy (8) due to their capability of driving neuronal differentiation *in vitro* (9).

Over the years, several studies focused on the identification of new molecular targets. However, although different experimental approaches have been used, including whole-genome sequencing, few somatic mutations in druggable pathways have been identified. Mutations in the anaplastic lymphoma receptor tyrosine kinase (*ALK*) are infrequent (<10% of sporadic NB). Mutations in the a-thalassemia/mental retardation syndrome X-linked (*ATRX*) gene are most common. However, they have not been identified in MYCN-amplified NB and the ATRX molecule does not appear to drive tumorigenesis (10, 11). Recurrent genetic alterations that, however, require further investigations, have been reported in the RAC–RHO pathway and in chromatin-remodeling genes At-rich interactive domain 1A (ARID1A) and 1B (ARID1B) (11–13). Due to the difficulty at targeting directly the MYCN transcription factor, the major oncogenic driver identified so far (14), new strategies currently aim to neutralize molecules involved in apoptosis, angiogenesis, invasion, or metastasis (5). Interestingly, the antibody-mediated targeting of the oncofetal differentiation antigen GD2 in combination with GM-CSF and IL-2 resulted in an improved outcome of patients and has been recently included in the standard care of high-risk NB (15). Unfortunately, pain, the major side effect of this therapy (16), as well as cytokine release syndrome and iridoplegia, limits its use; moreover, to date, only patients with minimal residual disease can benefit of this therapeutic approach (17). The success of the anti-GD2/GM-CSF/IL-2 therapy might depend on the FcγR-mediated activation of granulocytes (18) as well of other immune cell types such as macrophages and natural killer (NK) lymphocytes.

Among cytotoxic lymphocytes, NK cells represent the most potent anti-tumor effectors and represent promising weapons against aggressive tumors such as NB (19, 20). In addition, as recently demonstrated, they may also attack cancer stem cells (CSC), i.e. tumor cells with stem cell properties (21–23). Thus, in the past decade, several studies have been focused on the identification of the molecular mechanisms involved in the interaction between NB and NK cells in order to establish a convincing biological starting point for a novel NK cell-based immunotherapeutic approach.

## Natural Killer Cells and Neuroblastoma Recognition

Tumor cells express surface molecules that either switch off or switch on NK cell-mediated cytotoxicity. HLA class I molecules on tumors negatively regulate NK cell function by engaging immunoreceptor tyrosine-based inhibition motifs (ITIM)-bearing receptors (24) that include the inhibitory killer Ig-like receptors (KIRs, CD158), highly polymorphic clonally distributed receptors able to distinguish among different HLA-A, -B, and -C allotypes (25), and CD94/NKG2A heterodimers, specific for non-classical HLA-E (26). KIR and CD94/NKG2A are differently expressed in CD56^bright^ CD16^−^ and CD56^dull^ CD16^+^ NK cell subsets, which represent sequential stages of maturation (27, 28). While high numbers of CD56^bright^ CD16^−^, KIR^−^, IFN-γ producing cells are found in non-reactive lymph nodes and tumor sites, the majority of NK cells circulating in the blood are mature CD56^dull^ CD16^+^, KIR^+^, cytolytic (perforin^high^) cells (29). CD56^bright^ can progress to CD56^dull^ cells, which upon activation increase the cytotoxic and IFN-γ producing capabilities (28, 30).

Tumor cells switch on NK cell function by expressing at the cell surface non-MHC class I, “danger” molecules that are recognized by an array of activating NK receptors. These include both the FcγRIII (CD16) that mediates the antibody-dependent cytotoxicity (ADCC) and receptors that function in the absence of antibodies. These latter molecules mainly consist of the NKp46, NKp30, and NKp44 receptors, collectively termed natural cytotoxicity receptors (NCR), which are mostly NK-restricted, and NKG2D and DNAM-1 that are also expressed by T cell subsets (31). In the last decade, several groups dedicated many efforts to unveil the nature of the surface tumor ligands specifically recognized by the activating receptors. NKp46 still remains a receptor with “orphan ligand,” while NKp44 has been shown to recognize a novel isoform of the mixed-lineage leukemia (MLL5) protein (32) and NKp30 binds to B7-H6 molecules (33). The NKG2D and DNAM-1 (CD226) receptors recognize MICA/B and ULBPs or, PVR (CD155) and Nectin-2 (CD112), respectively (31). DNAM-1 shares the capability of recognizing PVR with tactile (CD96) (34) and TIGIT (35). All the ligands identified so far represent *de novo* expressed danger signals or molecules that are expressed in healthy cells and over-expressed upon tumor transformation. In most cases, the expression on tumors of multiple ligands leads to the engagement of different activating NK receptors that cooperate in triggering the anti-tumor cytotoxicity (31).

When both inhibitory and activating receptors are engaged by the specific ligands on potential targets, the function of ITIM-bearing inhibitory receptors dominates over activation. Thus, in an autologous setting NK lymphocytes spare healthy cells that express high, “protective” levels of HLA class I molecules while they kill tumors, such as NB, in which HLA class I expression is downregulated (36, 37). Moreover, NK cells kill allogeneic targets that express non-self MHC class I alleles. This might occur when two individuals are “KIR/KIR-ligand mismatched,” i.e., individual A is characterized by fully functional, educated NK cell subset(s) expressing KIR specific for HLA class I alleles that are missing in individual B. In the context of HLA-haploidentical hematopoietic stem cell transplantation (haplo-HSCT), the presence of a KIR/KIR-ligand mismatch in the donor versus recipient direction allows donor’s NK cell subsets to kill recipient’s (HLA class I^high^) residual leukemic blasts thus resulting into a graft-versus-leukemia effect (GvL), as well as mature DC and activated T lymphocytes, thus preventing graft-versus-host-disease (GvHD) and graft-rejection, respectively (38).

**Figure 1:**
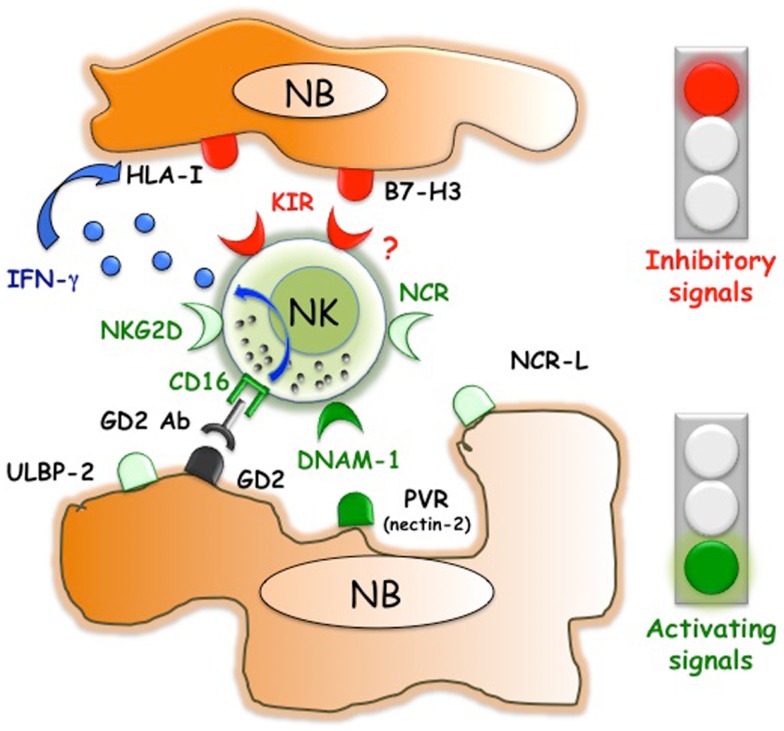
**Inhibitory and activating interactions between human NK cells and neuroblasts**. NB express ligands that are recognized by NK receptors with activating or inhibitory function. While DNAM-1/PVR interactions play a pivotal role in triggering NK cell-mediated killing, B7-H3 dampens NK cell function. NB usually lack or express low, non-protective levels of HLA class I molecules. However, therapeutic approaches such as the *in vivo* administration of anti-GD2 Abs could induce not only ADCC of NB but also the release of INF-γ, which upregulates the expression of the ligands for inhibitory KIR.

**Figure 2:**
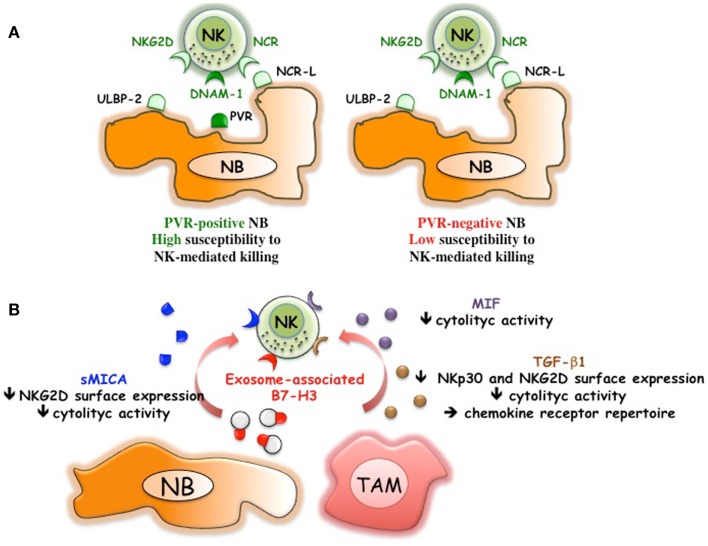
**NB-mediated mechanisms of escape from the NK-mediated immunosurveillance**. **(A)** Downregulation of PVR, ligand of DNAM-1 activating receptor, renders NB cells poorly susceptible to NK-mediated killing. **(B)** NB can release soluble MICA, exosome-associated B7-H3, immunomodulatory factors such as MIF and TGF-β1. TGF-β1 might be also released by tumor-associated macrophages (TAM) that display M2-like functional properties.

In NB, initial data were obtained by the use, as target cells, of *in vitro* established NB cell lines (39). NK cells displayed strong cytolytic activity against different NB cells and NCR were involved in the mechanisms leading to killing (39). The susceptibility of human NB cell lines to NK-mediated killing was validated in the context of a metastatic model set up in NOD/SCID mice. In this experimental setting, repeated infusions of IL-2 or IL-15 activated NK cells resulted in both an increased mean survival time of HTLA-230-bearing mice and reduced BM infiltration (40). Considering that long term cultured cell lines might be poorly representative of original tumors, more interesting data originated from *in vitro* studies that use neuroblasts, freshly purified from BM aspirate of stage 4 patients (36) (Figures 1 and 2). In this study, allogeneic activated NK cells killed neuroblasts isolated from patients although at a lesser extent as compared to the NB cell lines used as control. According to the absence or negligible expression of HLA class I molecules, the NK-mediated lysis of neuroblasts did not increase in the presence of anti-HLA class I mAb. Different molecular mechanisms responsible for the reduced HLA class I expression in NB cells have been elucidated. The immunohistochemical analysis of high-risk human NB showed different abnormalities in the antigen processing machinery, which include defects in the expression of immunoproteasomal subunits LMP2 and LMP7 and of transporters of antigen processing (TAP) (37). *In vitro* treatment of NB cells with IFN-γ induced up-regulation of HLA class I expression (37). Although decreasing their susceptibility to autologous NK cells, this up-regulation of HLA class I molecules could enhance T cell-mediated recognition. In this context, a restoration of killing mediated by antigen specific (MAGE3) cytolytic T cells was observed upon cotransfection of NB cell lines with IRF1 and NF-kB p65, HLA class I transcriptional activators that are also induced by INFγ and TNFα, respectively (41). It cannot be excluded that also *in vivo* neuroblasts could acquire/upregulate HLA class I expression. For example, this might occur in the context of transplantation or following the anti-GD2 antibody-mediated therapy that engages FcγR^+^ immune cells such as NK cells, which are capable of releasing high amounts of IFN-γ upon activation (Figure 1). This phenomenon was observed in a murine NB model, where recurrent tumors developed after an NK-dependent anti-tumor response induced by a humanized IL-2 immunocytokine targeted to GD2. In these mice, NB cells showed markedly enhanced MHC class I expression as compared with tumors growing in controls (42). The possible *in vivo* increase of HLA class I expression in NB cells could explain the benefit of a KIR/KIR-L mismatch in the NK versus NB direction (43–45).

Bone marrow-purified neuroblasts from different patients, although expressing in all instances low levels of HLA class I, displayed a great heterogeneity in terms of susceptibility to lysis by NK cells. The good or poor susceptibility to NK-mediated killing correlated with the presence or the absence of PVR expression on neuroblasts (Figures 1 and 2A)(36). Interestingly, neuroblasts lacking PVR expression were from patients in relapse, whereas those expressing PVR were from children at the onset of the disease, suggesting a possible correlation between PVR expression and NB status. The predominant role of DNAM-1/PVR interaction in NK-mediated killing is striking considering that, in most cases, killing of human tumors depends on the cooperation between various activating receptors (31). In this context, it is of note that none of the BM-purified neuroblast fractions expressed major adhesion molecules such as ICAM1 or LFA-3 (36). Thus, in NB the presence of PVR, originally described as an adhesion molecule, could become critical in rendering tumor cells susceptible to NK-mediated killing. Although PVR and nectin-2 are closely related molecules of the Nectin family, the presence/absence of nectin-2 on NB cells did not influence the susceptibility to NK-mediated killing. Nectin-2, which displays binding affinity to DNAM-1 similar to PVR (46), also shows homophilic recognition. This, by hampering its recognition by DNAM-1, may force the receptor to preferentially bind PVR (46). In line with the increased killing of PVR^+^ neuroblasts, mAb mediated masking of DNAM-1 significantly reduced the NK-mediated lysis of neuroblasts showing a pivotal role of DNAM-1 in NB cell killing. While a minor contribution of NKp46 and NKp30 activating receptors could be appreciated (36), consistent with the low expression of the specific ligands, NKG2D did not play a significant role in killing of BM-purified neuroblasts (Figure 1). The expression of NKG2D ligands has been also investigated in human primary NB and cell lines (47). *MICA, MICB*, and *ULBPs* transcripts were found in most tumors and cell lines. However, MICA surface expression was absent in primary tumors and present only in some NB cell lines. Importantly, the soluble form of MICA (sMICA) was detected in sera of most patients and in the culture supernatant of some cell lines and was shown to downregulate NKG2D surface expression in peripheral-blood T cells and decrease NK cell-mediated killing of MICA^+^ NB cells (47) (Figure 2B). MICB was detected exclusively in the cytosol of NB cells, either primary tumors or cell lines. ULBP-1 was rarely detected, ULBP-2 was expressed by approximately 50% of primary tumors and cell lines, and ULBP-3 was absent in primary tumors but was expressed in most NB cell lines (47).

A crucial role of the DNAM-1 receptor has also been reported in myeloid and lymphoblastic leukemia (48), ovarian tumors (49), and different murine tumor models (50, 51). In particular, it has been shown that in response to chemical carcinogens, DNAM-1-deficient mice develop more DNAM-1 ligand^+^ fibrosarcoma and papilloma cells than wt mice. Thus, the study of the molecular mechanisms responsible for the modulation of DNAM-1 ligands appears to be highly relevant. In this context, it has been shown that malignant RAJI lymphoma cells present hypermethylated PVR promoter-associated CpG islands. The epigenetic status was partially reversed by hypomethylating agents that led to upregulation of both transcription and surface expression of PVR (52). PVR up-regulation has been shown to be dependent on DNA-damage response (DDR)-dependent pathways that are activated by oxidative stress (53). Moreover, modulation of PVR surface expression might occur during infections. Different pathogen-derived stimuli such as LPS, poly I:C, and flagellin upregulated the surface expression of PVR (and nectin-2) in human DC (54) and in murine antigen-presenting cells (55) via the MYD88 and TRIF pathways. It has also been shown that expression of the human immunodeficiency virus type 1 (HIV-1) Vpr protein increases PVR levels in Jurkat T cells. This is due to activation of ATR kinase that triggers the DDR pathway and G2 arrest. Moreover, Vpr induces a PVR upregulation in HIV-infected CD4^+^ T lymphocytes that overcomes the PVR downregulation induced by the HIV Nef protein (56). A negative regulation of PVR surface expression has been observed during cytomegalovirus (CMV) infection resulting in ineffective NK cell-mediated killing of infected cells (57). The UL141 viral glycoprotein is responsible for PVR downregulation, since it retains the immature form of PVR in the endoplasmic reticulum (57). Recently, it has been shown that UL141 binds to PVR with high affinity (58). Considering the role of PVR in determining high susceptibility to the NK-mediated attack, up-regulation of PVR surface expression might represent an appealing strategy to strengthen the immune responses against tumors. However, the dark side of PVR function cannot be disregarded. Indeed, although data in NB are missing, PVR has been described as a positive regulator of cancer progression, since it is capable of enhancing tumor cell invasiveness and migration (59). Thus, further studies are required to understand whether the immunological favorable effects of PVR up-regulation might balance the induction of a more aggressive tumor phenotype.

A tumor-promoting role has been shown also for B7-H3, a surface molecule originally identified on NB thanks to the generation of a specific monoclonal antibody (M5B14) (60). Studies on different tumor histotype indicate that B7-H3 might drive tumor cell development by different molecular mechanisms. These include the capability of B7-H3 of reducing the effect of the chemotherapeutic-induced apoptosis in breast (61) and pancreatic carcinoma (62), as well as that of promoting migration and invasiveness (63, 64). The latter properties have been confirmed *in vivo* in an orthotopic model that showed that xenografted B7-H3 silenced glioma cells invaded significantly less into the surrounding brain tissue as compared to wt tumors. Moreover, B7-H3 expressed on glioblastoma cells negatively regulated NK cell-mediated cytotoxicity (64). These observations are consistent with previous data demonstrating that B7-H3 expression on BM-purified neuroblasts decreased NK cell-mediated killing (60) (Figure 1). Thus, different experimental approaches demonstrated that, in humans, B7-H3 might block the NK-mediated attack by interacting with a still unknown inhibitory NK receptor. A soluble form of B7-H3 (sB7-H3) that results from MMPs cleavage of the surface protein was detected in sera of tumor patients and appeared to correlate with tumor burden (65, 66). Moreover, B7-H3 is present in NB cell lines-derived exosomes (67). The exosome-associated form could inhibit NK cell function by engaging the B7-H3-specific inhibitory receptor (Figure 2B). Thus, B7-H3 is an interesting NB-associated molecule that combines immune-evading and tumor progression properties. This correlates with the negative prognostic value associated to high expression of B7-H3 in several tumor types (64, 66, 68–72). In particular, in primary NB (73), high B7-H3 surface intensity and percentage of positive cells correlated with poor event-free survival. Interestingly, the differences in event-free survival were observed also in patients with localized disease (stage 1–3), suggesting the need of a more careful follow-up/aggressive treatment of B7-H3^+^ low-risk patients. It should be mentioned that, in the mouse system, B7-H3 has been described as a “friend” in tumor immunology (74). In particular, intratumoral injection of an expression plasmid encoding mouse B7-H3 led to complete regression of 50% of tumors, which was mediated by NK and CD8^+^ T cells (75). It is to note that the mouse *B7-H3* gene codes for a molecule characterized by two Ig-like domains (2Ig-B7-H3) in the order IgV and IgC, while the predominant isoform in human tissues and cell lines is a longer molecule (4Ig-B7-H3) characterized by four Ig-like domains (IgV–IgC–IgV–IgC), which results from duplication of the exons encoding the IgV and IgC domains (76). The mouse B7-H3 has been shown to bind the TREM-like transcript 2 (TREML2, TLT-2) triggering receptor, which is expressed by myeloid cells, CD8^+^ T cells, and activated CD4^+^ T cells (77, 78). In humans, TREML2 does not appear to be a receptor of B7-H3 (79) and the predominant inhibitory role of B7-H3 strongly suggests the existence of a (still undefined) receptor that down-regulates NK cell function. However, the existence in the human system of specific receptors with opposite functions cannot be excluded. Indeed, human B7-H3 belongs to the B7 family that includes members such as B7–1 (CD80), B7–2 (CD86), B7-H2 (ICOS-L), B7-H1 (PD-L1), B7-DC (PD-L2), B7-H4 (B7S1, B7x), and BT3 (80), some of which represent specific ligands of receptors with either activating or inhibitory function. The *scenario* should become even more complex when considering that the B7-H3 inhibitory and activating receptors might be co-expressed in immune cells rather than expressed in different cell types/subsets.

## Tumor Escape Mechanisms in Neuroblastoma and Possible Novel NK Cell-Based Immunotherapeutic Approaches

In principle, downregulation of HLA class I surface expression allows NB to evade T cell-mediated attack operated by the host immune system (36, 37), while rendering tumor cells susceptible to NK cell-mediated recognition and killing (31). However, both the fast tumor progression in high-risk NB patients and the *in vitro* data highlighted the existence of additional escape mechanisms that dampen the NK cell-mediated anti-tumor activity as well. As discussed above, the lack of ligands for activating receptors such as PVR (36) (Figure 2A), MICA/ULBPs (36, 37), or adhesion molecules (36, 81) represents a strategy allowing NB cells to strongly reduce their susceptibility to NK-mediated killing. Thus, one could set up immunotherapeutic interventions aimed at upregulating the expression of these ligands. However, the role of PVR in tumor progression (59), as well as its expression in normal cells, should not be underestimated. In particular, DNAM-1 co-operates with NKp30 or NKp46 in the NK-mediated killing of autologous immature DC and unpolarized (M0) or M2-polarized macrophages, which express low, “non-protective” amounts of HLA class I molecules. Conversely, mature DC and M1-polarized macrophages express high levels of HLA class I molecules and are protected by the NK cell-mediated attack (54, 82). The NK-mediated selection of DC and macrophages with optimal antigen-presentation properties has been named “immunoediting” and results from the fine-tuning of inhibitory and activating signals provided by the ligands physiologically expressed by DC and macrophages. The forced over-expression of ligands for activating receptors, such as PVR, might dangerously alter the immunoediting process. Moreover, it should be taken into account that PVR is expressed in normal endothelium, where it plays a role in leukocyte extravasation (83, 84). Also in this case, up-regulation of PVR expression might overcome the HLA class I-mediated inhibitory signals and result in loss of self-tolerance toward autologous endothelium. On the other hand, it has been shown in primary glioblastoma that endothelial cells of proliferating tumor vessels express PVR at higher surface densities as compared to normal vessels (22). Moreover, several studied indicated that most tumor cells including CSC constitutively express levels of PVR higher than the normal counterparts (21). Although further studies are needed to dissect the possible side effects, therapeutic approaches focused on PVR might represent a chance in the treatment of highly aggressive tumors such as NB. Up-regulation of PVR in NB patients might increase the susceptibility to NK-mediated lysis of PVR^+^ NB and restored that of PVR^−^ NB. In this context, it is of note that the lack of PVR expression was detected in given BM metastatic neuroblasts (36) while no data are available on PVR expression in primary tumors. Thus, it cannot be excluded that PVR^−^ NB cells might originate because of the selection pressure occurring during the dissemination process and that primary tumors could contain PVR^+^ targetable neuroblasts. If so, a larger cohort of patients might benefit from PVR up-regulation therapies. Importantly, PVR might represent a powerful therapeutic target on both neuroblasts and NB-associated vessels. PVR has been originally identified as the receptor for the poliovirus, a highly contagious virus that only affects humans. As already mentioned in a study performed in a poliovirus-susceptible animal model, the oncolytic treatment by a novel attenuated poliovirus eradicated PVR^+^ NB cells without signs of paralysis (85).

**Figure 3:**
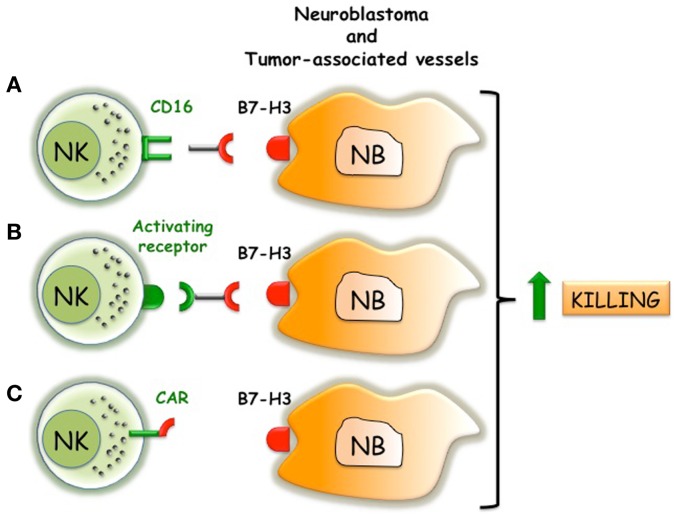
**Possible immunotherapeutic approaches that target B7-H3 and trigger NK cell function**. **(A)** Anti-B7-H3 antibodies induce ADCC of NK cells against NB. **(B)** Bi-specific antibodies that react with B7-H3 on NB and trigger activating receptors on NK cells. **(C)** NK cells engineered with B7-H3-specific CAR that transduce activating signals via CD3 chain.

B7-H3 might represent an additional attractive molecular target in NB. Beside its tumor-promoting properties, human B7-H3 represents a shield protecting HLA class I ^low/−^ neuroblasts from the NK cell-mediated attack (60, 64) (Figure 1). B7-H3 is expressed in normal endothelium, but is strongly upregulated in tumor-associated vasculature (86). Encouraging results derived from the first in-human intrathecal injection of radioiodinated anti-B7-H3 Ab (following surgery, craniospinal irradiation, and chemotherapy) in 21 NB patients with recurrent CNS metastasis (87). Seventeen patients remained CNS disease free and had a median survival time (33 months) significantly better than patients treated with standard protocols (6.6 months) (88). Anti-B7-H3 antibodies, possibly delivered using lipid-based (liposome) formulations (89–91), might represent a suitable option to target tumoricidal compounds to NB and to increase immune responses (Figure 3). Indeed, acting on different cell types, it might simultaneously block invasiveness/migration of tumor cells, destroy tumor-associated vessels, and strength the activity of NK cells. Moreover, anti-B7-H3 antibodies might induce NK cell-mediated ADCC against NB and destroy the B7-H3/inhibitory receptor interactions. An alternative approach to trigger NK cell function might consist in the use of bi-specific antibodies reacting with B7-H3 (on tumor cells) and activating receptors (on NK cells). In addition B7-H3 might be considered as a molecule alternative to GD2 in CAR-based immunotherapy (Figure 3). CAR are chimeric antigen receptors, formed by tumor-specific Ab single chain Fv fragments (scFvs) genetically fused through a transmembrane domain to the CD3ζ chain, which are transfected into cytolytic lymphocytes (92, 93). The use of CD19-specific CARs resulted in a significant clinical benefit in two children with B-cell precursor acute lymphoblastic leukemia (94). The NK92 cell line engineered to express the GD2-specific CAR showed increased cytolytic activity against NB cell lines and primary NB cells (95). Moreover, T lymphocytes equipped with GD2-specific CAR are being used in preclinical and phase I studies with clinical benefit in NB patients (96, 97). Essential requirement in CAR-based immunotherapy is the selection of an adequate tumor surface antigen with a documented tumor-promoting role (92). B7-H3 satisfies this criterion, thus representing a suitable candidate in CAR-based immunotherapy. Moreover, while GD2-specific CAR target a molecule that is easily shed by the NB cell surface (98), B7-H3-specific CAR would target a stable type I transmembrane surface molecule expressed by NB cells and NB-associated vessels. Infusion of engineered and/or activated (by cytokine or DC) (99) NK cells might be helpful in adoptive immunotherapeutic approaches of high-risk NB patients receiving autologous (5) or haplo-HSCT. Indeed, a gap of several weeks exists between the infusion of CD34^+^ stem cells and the appearance in peripheral blood of fully “armed” mature NK cells (38). During the early post-transplant period, lack of NK-mediated immunosurveillance could favor re-growth of residual NB cells escaping the preparative regimen to the allograft, this causing tumor relapses. Haplo-HSCT, which represents a new standard therapy in the cure of adult and pediatric leukemic patients, could also increase the event-free survival of NB patients (45). Recently, a novel transplant strategy based on negative depletion of both α/β T lymphocytes and of CD19+ B-cells has been proposed for pediatric hematological disorders. This approach leaves in the graft an array of immune cells with anti-tumor properties, including mature armed donor-derived alloreactive NK cells and γ/δ T cells (38). Also in this type of transplant that preserves donor mature NK cells in the graft, engineered NK cells might support the anti-tumor responses. In the perspective of *in vivo* NK-based adoptive immunotherapies as adjuvant in different transplant strategies, recent studies optimized the *in vitro* expansion and activation of NK cells that preserved the anti-NB activity (100–103).

In addition to the escape mechanisms described above (47, 60, 104), NB cells produce soluble factors such as MIF (105, 106) and TGF-β (107) capable of dampening NK cell activity (108, 109) (Figure 2B). Initial data showed that *in vitro* conditioning of NK cells with rTGF-β1 resulted in downregulation of NKp30 and NKG2D activating receptors and in significant reduction of NK-mediated killing (109). Different studies confirmed *in vivo* the role of TGF-β in regulating NKG2D expression and function in NK cells (110, 111). Although knockout mice lacking TGF-β1 or TGF-β2 showed distinct phenotypes suggesting that each isoform could also have specific, non-overlapping functions (112), *in vitro* high concentrations of both rTGF-β1 and rTGF-β2 (>10 ng/ml) showed similar capability of inhibiting the expression of the activating NK receptors (113). It has been shown that NB cell lines released low/medium amounts of TGF-β1 (<3 ng/ml), which were unable to significantly downregulate NKp30 and NKG2D expression while clearly modulated the chemokine receptor repertoire of NK cells (113). Interestingly, non-MYCN-amplified NB cells produced amounts of TGF-β1 (and several other soluble factors) significantly higher than MYCN amplified cells (113). NB-derived TGF-β1 upregulated the expression of CXCR4 and CXCR3 in all NK cells and downregulated that of CX3CR1 in the CD56^dim^ subset. Notably, a similar altered chemokine receptor repertoire was observed in peripheral-blood NK cells of stage 4 NB patients resulting in the appearance of a CX3CR1^low^ CD56^dim^ NK cell population. In patients, also a tendency to CXCR3 upregulation on CD56^bright^ NK cells was appreciated. No significant differences in CXCR4 expression were detected in NK cells from NB patients as compared to healthy donors. This might be due to a general difficulty in detecting *in vivo* CXCR4 expression, which in particular in the BM, might be fully occupied by the CXCL12 (SDF1) ligand (114). In NK cells from NB patients, also a significant CXCR1 down-modulation was observed, that, however, was TGF-β1-independent (113). Thus, *in vivo*, NB cells can release TGF-β1 and other, still unknown soluble factor(s) that profoundly affect the expression of a number of chemokine receptors that play pivotal roles in NK cell BM homing, egress, interaction with endothelium, and recruitment into peripheral tissues. High concentrations of TGF-β1 might locally exert a paracrine effect on tumor-associated NK cells that might decrease their anti-tumor activity by downregulating NKp30 and NKG2D expression. In this context, it has been shown that CD56^bright^, immature, poor cytolytic NK cells represent the major subset present in tumor tissues (115, 116). The above data suggest that TGF-β1 antagonists and/or proinflammatory cytokines capable of overcoming the modulatory effect of TGF-β might represent a reasonable adjuvant therapy in the cure of different tumors, including NB. In this context, mouse models showed that use of IL-2 targeted to GD2 is associated with increased infiltration of NK (and CD8+ T cells) in subcutaneous NB (117).

Novel immunotherapeutic approaches should also consider the complexity of tumor microenvironment that is populated by different immune cell types that could be endowed with immunomodulatory functions. These cells include fibroblasts (118), regulatory T cells (Treg), myeloid derived suppressor cells (MDSC) (119), and tumor-associated macrophages (TAM) (120). Different NB mouse models demonstrated the immunosuppressive and tumor-promoting role of Treg (120) and MDSC (121). To date, however, data on their presence within the human NB tumor and on their clinical relevance are still scarce. A recent study showed that metastatic NB have higher infiltration of TAM than locoregional tumors, and that metastatic tumors in patients ≥18 months have higher expression of inflammation-related genes than those in patients <18 months (122). TAM are characterized by an “M2-like” functional phenotype and exert tumor-promoting and immunoregulatory properties, including the release of TGF-β1 (120) (Figure 2). M2-polarized macrophages still present a functional plasticity and they can be recommitted toward an M1-like tumor suppressing, immunostimulatory phenotype. In particular, it has been shown that microbial products such as LPS or BCG reverted their functional phenotype toward M1, which released immunostimulatory cytokines (IL-12, IL-18) and induced strong activation of autologous NK cells. NK cells up-regulated CD69 and CD25 (that associates with CD122 to form the high-affinity receptor for IL-2), expressed CCR7 (a chemokine receptor involved in homing of NK cells to secondary lymphoid organs), increased the anti-tumor cytolytic activity, and released high amounts of IFN-γ (82). Interestingly, it has been shown that a subset (30–40%) of M2 express a membrane-bound form of IL-18 (mIL-18) that is released upon TLR stimulation (123, 124). This soluble form of IL-18 (sIL-18) by acting in close cell-to-cell contact is crucial for both IFN-γ release and expression of CCR7 by NK cells. These data suggest that reconverting the immunosuppressive TAM phenotype using apathogenic TLR ligands might represent an additional immunotherapeutic approach to fully activate immature tumor-associated NK cells.

## Conclusion

Progress in the understanding of NB cell biology will allow a more accurate stratification of patients, thus reducing toxic side effects of aggressive therapy in low-risk patients. High-risk patients who currently have a dismal prognosis could benefit from multidisciplinary therapeutic protocols that include novel NK cell-based immunotherapeutic strategies. The latter will take advantage of our knowledge about the presence/absence of NB-associated ligands interacting with activating/inhibitory receptors expressed by NK cells. Moreover, it should also take into account the multiple immunomodulatory strategies set up by NB and various immune cell types to impair the recruitment and activation of NK cells in the tumor microenvironment. The encouraging results emerged from haploidentical hematopoietic cell transplantation in pediatric hematological malignancies, might strongly motivate a re-evaluation of transplant approaches in the therapy of high-risk NB patients.

## Conflict of Interest Statement

Alessandro Moretta is a founder and shareholder of Innate Pharma (Marseille, France). The remaining authors declare no conflicts of interest.
